# Hospital Note

**Published:** 1903-04

**Authors:** 


### HOSPITAL NOTE.

---

THE Marine Hospital of Buffalo, which has been provided for by Congress, will be located on upper Main Street, between Providence Retreat and the Institute for the Deaf and Dumb. It will occupy three acres, purchased from these two institutions, which will be an appropriate site for an imposing structure, such as is contemplated. Work upon the building will begin at an early day, or as soon as the appropriation therefor becomes available.

